# Language Artificial Intelligence Models as Pioneers in Diagnostic Medicine? A Retrospective Analysis on Real-Time Patients

**DOI:** 10.3390/jcm14041131

**Published:** 2025-02-10

**Authors:** Azka Naeem, Omair Khan, Syed Mujtaba Baqir, Kundan Jana, Prem Shankar, Avleen Kaur, Morad Zaaya, Fatima Sajid, Fizza Mohsin, Marlon Rivera Boadla, Aung Oo, Victor Wong, Momna Noor, Samar Pal Singh Sandhu, Kseniya Slobodyanuk, Vijay Shetty, Aaron Z. Tokayer

**Affiliations:** 1 Maimonides Medical Center, Brooklyn, NY 11219, USA; sbaqir@maimo.org (S.M.B.); kjana@maimo.org (K.J.); pshankar@maimo.org (P.S.); mzayaa@maimo.org (M.Z.); fsajid@maimo.org (F.S.); fmohsin@maimo.org (F.M.); marorivera@maimo.org (M.R.B.); aoo@maimo.org (A.O.); viwong@maimo.org (V.W.); mnoor@maimo.org (M.N.); sasandhu@maimo.org (S.P.S.S.); vshetty@maimo.org (V.S.); atokayer@maimo.org (A.Z.T.); 2 Department of Internal Medicine, Division of Rheumatology, University of Nebraska Medical Center, Omaha, NE 68198, USA; okhan@unmc.edu; 3 Department of Gastroenterology, SUNY Upstate Medial University, Syracuse, NY 13210, USA; kauravl@upstate.edu; 4 NYU Langone Health, New York, NY 10016, USA; ks1538@nyu.edu

**Keywords:** artificial intelligence, cardiology, gastrointestinal, emergency department

## Abstract

**Background/Objectives:** GPT-3.5 and GPT-4 has shown promise in assisting healthcare professionals with clinical questions. However, their performance in real-time clinical scenarios remains underexplored. This study aims to evaluate their precision and reliability compared to board-certified emergency department attendings, highlighting their potential in improving patient care. We hypothesized that board-certified emergency department attendings at Maimonides Medical Center exhibit higher accuracy and reliability than GPT-3.5 and GPT-4 in generating differentials based on history and physical examination for patients presenting to the emergency department. **Methods:** Real-time patient data from Maimonides Medical Center’s emergency department, collected from 1 January 2023 to 1 March 2023 were analyzed. Demographic details, symptoms, medical history, and discharge diagnoses recorded by emergency room attendings were examined. AI algorithms (ChatGPT-3.5 and GPT-4) generated differential diagnoses, which were compared with those by attending physicians. Accuracy was determined by comparing each rater’s diagnoses with the gold standard discharge diagnosis, calculating the proportion of correctly identified cases. Precision was assessed using Cohen’s kappa coefficient and Intraclass Correlation Coefficient to measure agreement between raters. **Results:** Mean age of patients was 49.12 years, with 57.3% males and 42.7% females. Chief complaints included fever/sepsis (24.7%), gastrointestinal issues (17.7%), and cardiovascular problems (16.4%). Diagnostic accuracy against discharge diagnoses was highest for ChatGPT-4 (85.5%), followed by ChatGPT-3.5 (84.6%) and ED attendings (83%). Cohen’s kappa demonstrated moderate agreement (0.7) between AI models, with lower agreement observed for ED attendings. Stratified analysis revealed higher accuracy for gastrointestinal complaints with Chat GPT-4 (87.5%) and cardiovascular complaints with Chat GPT-3.5 (81.34%). **Conclusions:** Our study demonstrates that Chat GPT-4 and GPT-3.5 exhibit comparable diagnostic accuracy to board-certified emergency department attendings, highlighting their potential to aid decision-making in dynamic clinical settings. The stratified analysis revealed comparable reliability and precision of the AI chat bots for cardiovascular complaints which represents a significant proportion of the high-risk patients presenting to the emergency department and provided targeted insights into rater performance within specific medical domains. This study contributes to integrating AI models into medical practice, enhancing efficiency and effectiveness in clinical decision-making. Further research is warranted to explore broader applications of AI in healthcare.

## 1. Introduction

GPT-3.5 is a state-of-the-art language artificial intelligence (AI) model that can generate human-like responses to human-devised prompts. It has also been used by medical students for assistance in learning medical terminology and subjects. An article published by the American Medical Association revealed that the generative AI chatbot ChatGPT has been found to be capable of passing the United States Medical Licensing Exam (USMLE) at 60% accuracy [1]. Ever since, its potential has been explored in medicine, as it can assist physicians in providing up-to-date information on specific medical conditions, symptoms, and treatments.

According to a prior study, tools like ChatGPT could be incorporated into medical education and ultimately into clinical decision-making [2]. GPT-3.5 has been known to assist in creating electronic health records, summarizing patient histories, and even generating patient-specific differential diagnoses and treatment plans. A pilot study executed by Takanobu et al. in February 2023 demonstrated that the diagnostic accuracy of ChatGPT-3.5 to form a list of 10 valid and correct differential diagnoses was almost 90% [3].

The recently introduced GPT-4 has new features including multimodal input/output, increased capacity for multitasking, enhanced accuracy, and safety. Furthermore, an article published in March 2023 is one of the pioneers discussing the role of ChatGPT-4 in medical scenarios. The article discusses the capabilities of ChatGPT-4 in the medical field, including its ability to generate medical notes based on physician-patient conversations, answer medical questions, and provide useful responses to typical medical situations. While ChatGPT-4’s medical knowledge is impressive, the article notes the need to address its potential mistakes and omissions in future deployments [4].

A prior study review has explored the utility of diagnosis, classification, and prognostication of specific diseases such as pulmonary hypertension [5]. However, not a lot of studies have focused on diagnosis in real-time hospital settings.

Keeping this in mind, the aim of our study is to evaluate the accuracy of GPT-3.5 and GPT-4 in the medical field by comparing the top 5 differential diagnoses generated by AI, with those documented by the ED attending physicians and eventual discharge diagnosis. We further want to compare this with specific subspecialty complaints pertaining to the cardiovascular and gastrointestinal systems. Finally, we also want to compare GPT-3.5 and GPT-4 in this regard, to assess if one is superior to the other in formulating accurate differential diagnoses.

## 2. Methods and Materials

**Figure 1:**
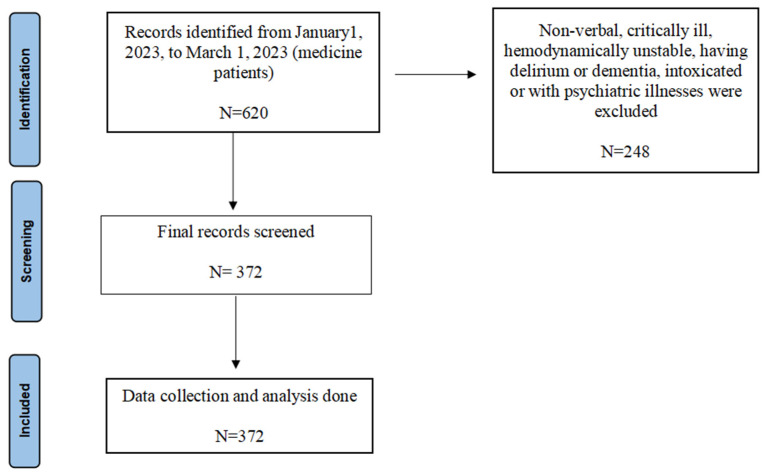
Flowchart showing the selection of the patients.

This manuscript has been prepared in accordance with the strengthening of the reporting of observational studies in epidemiology (STROBE). This was an accuracy and inter-rater reliability study performed using real-time patients presenting to the emergency department at Maimonides Medical Center. The study protocol was reviewed and approved by the institutional review board to ensure patient privacy and confidentiality and ensuring adherence to ethical principles and guidelines. The records of eligible patients admitted to the Internal Medicine department via the Emergency Department (ED) from 1 January 2023 to 1 March 2023 were reviewed to gather pertinent data. This included demographic details, presenting symptoms, past medical history, length of hospital stay, discharge diagnosis, and differential diagnoses recorded by ED attendings. The extracted data were reviewed and checked for completeness and accuracy. Any discrepancies or missing data were removed. Patients had to be between 18 and 65 years of age, hemodynamically stable, and verbal, to be able to express their complaints, to be included in the study. Patients who were critically ill, having delirium or dementia, intoxicated, or with psychiatric illnesses, were excluded (Figure 1). Any identifiers like the patient’s name, MRN, or date of birth were not provided to ChatGPT to maintain confidentiality. A standard template was provided to ChatGPT to generate differential diagnoses. The following template was used,

“___ year old male/female with PMH ____ presents to the ED with complaints of ____ going on for ____ days. Vital shows showed _______. On exam, the patient noted to have _____. Please provide us with the most relevant top 5 differential diagnosis based on this information”.

Using machine learning techniques, AI algorithms of both ChatGPT-3.5 and GPT-4 generated a list of the top 5 possible differential diagnoses. These differentials were compared with those generated by the emergency department attending on presentation to the hospital as well as the medicine attending at the time of discharge on the discharge summary, respectively. The list of differentials generated by the two models was compared among themselves as well (ChatGPT-3.5 vs. ChatGPT-4).

## 3. Statistical Analysis

To assess the accuracy and precision of the differentials generated by ED attendings, ChatGPT-3.5, and ChatGPT-4, a comprehensive statistical analysis was conducted. Accuracy, defined as the proportion of correctly identified cases out of the total cases, was calculated separately for each rater, in comparison with the gold standard, i.e., discharge diagnosis. This involved determining the number of true positives (cases correctly identified as positive) and true negatives (cases correctly identified as negative) and dividing this sum by the total number of cases. The precision of the differential diagnoses generated by ED attendings, Chat GPT 3.5, and Chat GPT 4 was evaluated using Cohen’s kappa coefficient and the Intraclass Correlation Coefficient (ICC). For precision, Cohen’s kappa was calculated separately for each pair of raters (Table 1). Cohen’s kappa coefficient provides a measure of agreement between two raters while accounting for the agreement occurring by chance (Table 2). 

Additionally, the Intraclass Correlation Coefficient (ICC) was computed to assess the consistency of the differential diagnoses across all three raters. ICC quantifies the degree of agreement among multiple raters for continuous or ordinal data, such as differential diagnoses. The value of an ICC can range from 0 to 1 with 0 indicating no agreement among raters and 1 indicating perfect agreement among raters. 

## 4. Results

Patient records from 1 January 2023 to 1 March 2023 were examined, gathering demographic details, medical history, and diagnosis, recording by board-certified emergency room attendings. The most common reasons for exclusion from the study were being delirious/altered level of consciousness, critically ill requiring intubation or vasopressors, and being intoxicated. In total, 372 patients were deemed appropriate to be included in the study on the premise that they were able to express their presenting complaints. Demographic and clinical characteristics of the study population are summarized in Table 3. The mean age of patients included in the study was 49.12 years. Gender distribution revealed that 213 individuals (57.3%) were male, while 159 individuals (42.7%) were female. Chief complaints varied, with fever/sepsis being the most common (24.7%), followed by gastrointestinal complaints (17.7%) and cardiovascular complaints (16.4%). Respiratory complaints accounted for 12.4% of cases, while neurological complaints were reported in 7.3% of cases. Falls, pain, and miscellaneous issues were less common, comprising 1.9%, 3.0%, and 16.7% of the reported chief complaints, respectively. These findings provide insights into the distribution and nature of the presenting symptoms among the study population.

In our investigation, we designated the final diagnosis at discharge as the gold standard. When assessing accuracy in comparison to this gold standard, Rater 3 (ChatGPT-4) demonstrated the highest accuracy at 85.5% (Table 4). Rater 2 (ChatGPT-3.5) and Rater 1 (Emergency Department attending) exhibited accuracies of 84.6% and 83%, respectively. Consequently, Rater 3 emerges as the most accurate among the assessed raters.

Regarding assessment of reliability among the raters, a moderate level of agreement, indicated by a kappa value of 0.7, was observed between Rater 2 and Rater 3 (Table 5). However, this level of agreement was found to be statistically insignificant, with a *p*-value greater than 0.01. Conversely, when comparing Rater 1 with either Rater 2 or Rater 3, a notable decrease in agreement was noted. Specifically, Rater 1 and Rater 3 exhibited minimal agreement, with a kappa value of 0.31 and a *p*-value less than 0.01. Similarly, minimal agreement was observed between Rater 1 and Rater 2, with a kappa value of 0.23 and a *p*-value less than 0.01.

To assess the reliability of all the raters altogether, an inter-class correlation coefficient (ICC) was used (Table 6). The value of an ICC can range from 0 to 1, with 0 indicating no agreement among raters and 1 indicating perfect agreement among raters. The ICC values of 0.41 calculated in our analysis suggest a poor level of agreement among raters, which was statistically significant with a *p*-value < 0.01 as shown in Table 6. 

## 5. Stratified Analysis

Stratified analysis was performed to further compare the accuracy and reliability of the three raters for the complaints related to gastrointestinal and cardiovascular systems.

The results of the stratified analysis (Table 7) revealed that overall, the three raters were more accurate in determining differential diagnoses for the complaints related to the gastrointestinal system than the cardiovascular system. Among the three raters, for gastrointestinal system complaints, Rater 3 was the most accurate (87.5%), followed by Rater 2 (85.22%), and Rater 1 was the least accurate (84%). For complaints related to the cardiovascular system, rater 2 was the most precise (81.34%) followed by rater 3 (80.23%), and again rater 1 was the least accurate (77.9%).

When assessing for reliability using the Cohen’s kappa test, there was an overall increased level of agreement among the raters when evaluating the differential diagnoses for cardiovascular system complaints rather than the gastrointestinal system presenting complaints (Table 8). Specifically, minimal agreement was observed between rater 1 and 2 (Kappa 0.32), and a weak level of agreement between rater 1 and 3 (Kappa 0.51) for cardiovascular complaints. However, a moderate level of agreement was noted between rater 2 and 3 (Kappa 0.74). Conversely, for gastrointestinal complaints, minimal agreement existed between rater 1 and 2 (Kappa 0.17) and rater 1 and 3 (Kappa 0.12), which was statistically insignificant. Notably, there was a moderate level of agreement between rater 2 and 3 (Kappa 0.71).

The Fleiss’ kappa test was utilized to assess the overall agreement between all raters collectively (Table 9). A moderate level of agreement was observed among all three raters concerning the differentials related to cardiovascular complaints. Conversely, only a fair level of agreement was noted among the raters regarding gastrointestinal complaints.

## 6. Discussion

We conducted a comprehensive analysis to determine the potential of ChatGPT and its use in the healthcare setting as a diagnostic tool. Our study employed real-time patients presenting to the emergency department of Maimonides Medical Center to evaluate the accuracy and reliability of differential diagnoses generated by both AI algorithms (ChatGPT-3.5 and GPT-4) and medical professionals (Emergency Department attendings and medicine attendings). Our study revealed that ChatGPT-4 had the highest accuracy compared to the other raters, overall as well as when stratified by disciplines. Additionally, the accuracy was greater for gastrointestinal complaints. However, compared to cardiovascular complaints, there was less agreement amongst all raters for gastrointestinal complaints.

A prior study evaluated ChatGPT-3.5, ChatGPT-4, and physicians regarding their top differentials and diagnostic accuracy within a list of differentials based on clinical case scenarios from case reports published on PUBMED [6]. This study portrayed a greater than 80% diagnostic rate of ChatGPT-4 within the top 5 and top 10 list of differentials. In congruence with the above, our study also generated an analysis of ChatGPT-3.5, ChatGPT-4, and physicians but there were some salient differences. Our study unveiled that ChatGPT-4 demonstrated the highest diagnostic accuracy, closely followed by ChatGPT-3.5 and the Emergency Department attending. These findings suggest that the accuracy of the AI models is comparable to the ED attending when it comes to generating the top five differential diagnoses in a real-time clinical scenario. However, the study by Hirosawa et al. was limited in terms of case acuity and case numbers. Since these were not randomly sampled and were chosen based on a certain criterion, they were not generalizable. Moreover, it was based on physicians generating differentials by reading clinical vignettes in relatively controlled environments. Our study was based on cases coming to the emergency department leading to broader differentials that make the comparison more effective. Furthermore, the physician’s differentials were based on the first-hand encounter with the patient based on medical history and physical examination alone with no details about laboratory or radiological investigations. This allowed us to generate a more pragmatic analysis since it was a comparison of limited information exposure to ChatGPT and the physician. This also helped us to determine the important utility of ChatGPT in more emergent situations, facilitating physicians in settings of high patient census. However, minimal agreement was found between ED physicians and ChatGPT-3.5 or ChatGPT-4, highlighting challenges in consensus among assessments.

Moreover, another study evaluated the performance of ChatGPT-3.5 in terms of differential diagnosis and correct diagnosis within the differential list of common clinical scenarios generated by physicians [7]. This was compared to the rate of correct diagnosis by physicians. This study revealed a 93.3% rate of diagnosis amongst the list of differential diagnoses which although inferior to that of the physicians, still demonstrated the importance of exploring the utility of AI Bots in helping physicians make the correct diagnoses. Unlike the study by Hirosawa et al., this study only used history and physical examination as the input, so it analyzed ChatGPT-3.5’s computation skills more comprehensively with limited information. However, unlike our study, they did not have a comparison of ChatGPT-4 and was also based on created clinical scenarios rather than real clinical cases, thus representing a unique opportunity to evaluate the performance of AI models within clinical scenarios. While the study by Hirosawa included important common complaints as reported in the literature, it was still a restriction in terms of the variety of presenting complaints that are encountered in a real-life setting.

A study by Ueda et al. explored ChatGPT-4’s performance on the New England Journal of Medicine image quiz, covering clinical vignettes from different medical specialties [8]. ChatGPT was evaluated with and without multiple choice options, achieving 98% and 89% accuracy, respectively. Even though this study tested one additional aspect, which is image interpretation, the clinical vignettes were briefly structured and not entirely applicable to real-life scenarios in terms of variety and presentations. Furthermore, there was no comparison with physicians which is important as that will help us determine in the long run if the use of AI can enhance medical care and ultimately be used as a diagnostic tool. 

A study compared ChatGPT-4’s performance with that of radiologists from different backgrounds to evaluate diagnostic accuracy based on the patient’s history and imaging findings. This was based on 30 complex neuroradiological cases [9]. The results revealed that ChatGPT’s performance was inferior to that of the radiologists in all three tiers of experience. However, this study had several limitations. They had a very small sample size, resulting in the plummeting power of the study, which translates into a lack of statistical significance. Furthermore, since these results are from one specific subspecialty, they cannot reflect ChatGPT-4’s overall performance. Moreover, these cases were evaluated by physicians in a relatively controlled environment; hence, it would not form an appropriate comparison with ChatGPT if it were to be used with real case scenarios. Lastly, the definition of complex was not specified thus introducing subjectivity when evaluating performance. Our study addresses these limitations by incorporating a substantial sample size of 372 individuals with a wide range of presenting complaints across various body systems, enhancing the study’s practicality and generalizability.

Barash et al. evaluated Chat GPT’s ability to recommend which patients needed further radiologic evaluation [10]. This was tested on 40 cases comprising various pathologies including pulmonary embolism, obstructing kidney stones, acute appendicitis, diverticulitis, small bowel obstruction, acute cholecystitis, hip fracture, and testicular torsion. ChatGPT-4 showed promising results in terms of appropriate radiology referrals.

An innovative aspect of our study was implementing a stratified analysis to assess the accuracy and reliability of the three evaluators, specifically for presenting complaints related to the gastrointestinal and cardiovascular systems. This approach allowed for a targeted evaluation of rater performance within these critical medical domains. In the stratified analysis, the three raters demonstrated higher accuracy in diagnosing gastrointestinal complaints than cardiovascular complaints. ChatGPT-4 demonstrated the highest accuracy for gastrointestinal complaints, while ChatGPT-3.5 exhibited the highest precision for cardiovascular complaints. Overall, there was greater agreement among raters for cardiovascular diagnoses than gastrointestinal diagnoses, as indicated by Cohen’s kappa and Fleiss’ kappa tests. It underscores the importance of a detailed history and analysis of gastrointestinal complaints. The observed variation could be attributed to the diverse range of non-specific complaints linked to gastrointestinal issues, in contrast to the cardiovascular system, which presents more distinct and limited complaints.

Recently studies have explored the utility of AI in cardiogenic shock using certain therapeutic devices such as the impella since they serve as sources of real-time data [11]. The study published recently [12] highlight the potential of AI integration in Impella-supported mechanical circulatory support (MCS) to enhance clinical decision-making. These studies showed that AI could improve patient care by optimizing therapy pathways, predicting outcomes, refining device implantation, and managing haemocompatibility-related issues. It is understandable that using AI in such high-risk situations requires maximizing machine learning with a wide range of data and careful interpretation of results. Our study portrays the utility of clinical decision-making in real-life situations which can be taken as an initiation point to further integrate AI in applications such as the one quoted above.

Our study has several strengths. It was based on real-time patients presenting to the emergency department of a major referral center. This gave our study an adequate sample size, leading to a practical analysis and comparison between our AI bots and physicians. Moreover, since we derived the real-time evaluation of cases at the point of first contact with a physician, using their differentials based on history and examination findings, our study gave a pragmatic analysis—how ChatGPT’s potential can be tapped for real-time evaluation compared to physicians in an uncontrolled environment. The physicians were chosen randomly, so the results were not biased toward the experience and expertise of physicians. Our study compared both ChatGPT-3.5 and 4’s performance, with results revealing that ChatGPT-4 was superior, which is compatible with prior studies.

There were some limitations in our study. This was a single-center study therefore the results cannot be extrapolated to other healthcare scenarios and environments. The physicians were from a single department. Even though the emergency department deals with all initial presentations, the results may not apply to GPT’s performance across all specialties. Since predominantly the pathologies pertained to cardiovascular, digestive system, and infectious, the results cannot apply to other disciplines. Various prior studies used structured cases from published reports or those made by physicians. Therefore, ChatGPT received consistent input. Since the initial presenting complaint of the patient was used in our case, the input for the same disease may not have been consistent and would have influenced the differentials derived by ChatGPT. Furthermore, our study did not account for physician experience which means that Chat GPT may or may not be accurate when compared to more experienced attending physicians so the results cannot definitely state that AI bots are superior to the evaluation done by the physicians. This variability interferes with the comparison between clinicians and AI models. Since there was moderate to weak agreement among raters, as shown by Cohen’s Kappa and Intraclass Correlation Coefficient (ICC), this disagreement in the practical setting brings about a major conflict between AI and clinicians on the topic of medical decisions—potentially initiating ethical dilemmas.

Future studies are needed to evaluate this application of AI with more generalizability such as including multicenter data which will also increase the spectrum of pathologies with reference to disciplines. Furthermore, accounting for physician experience would facilitate a more valid comparison. Specific documentation and analysis of mistakes or misdiagnoses by physicians and AI would help in patient care and understanding the accurate feeding of AI algorithms.

Regardless of the limitations, the study portrays that AI has a lot of potential to help in emergency care, especially when it comes to making time-sensitive diagnoses in high-pressure environments. The moderate levels of agreement, if anything, underscore the importance of careful interpretation of results generated by AI models. Making AI work well with healthcare workflows is a major challenge, especially when there are differences between diagnoses made by AI and those made by a doctor, which is something we have to eventually become comfortable with given the fast integration of technology in healthcare.

In conclusion, the study gives us a lot of useful information about how ChatGPT-3.5 and GPT-4 work in real-life clinical settings, but we should still think of its results as preliminary. A more comprehensive and systematic methodology is required to thoroughly evaluate the role of AI in diagnostic medicine. The incorporation of AI technologies such as ChatGPT-4 into medical practice should be undertaken with caution, emphasizing thorough validation, ethical supervision, and collaboration between AI and human physicians.

In summary, ChatGPT-3.5 and ChatGPT-4 were found to be fairly accurate in generating differentials which warrants more studies for their use in aiding physicians in improving patient safety and patient outcomes. Furthermore, the stratified analysis by discipline teaches us an important lesson on detailed exploration of gastrointestinal complains.

## Figures and Tables

**Table 1:** Description of raters.

Gold Standard	Discharge Diagnosis
Rater 1	Emergency department attending
Rater 2	Chat GPT 3.5
Rater 3	Chat GPT 4

**Table 2:** Interpretation of Cohen’s Kappa.

Value of Kappa	Level of Agreement
0–0.20	None
0.21–0.39	Minimal
0.40–0.59	Weak
0.60–0.79	Moderate
0.80–0.90	Strong
Above 0.90	Almost perfect

**Table 3:** Demographic and clinical characteristics of the patients admitted.

Characteristics	Categories	N (%)
Mean Age		49.12
Gender	Males	213 (57.3%)
	Females	159 (42.7%)
Chief Complaint	Gastrointestinal	66 (17.7%)
	Cardiovascular	61 (16.4%)
	Fever/Sepsis	92 (24.7%)
	Respiratory	46 (12.4%)
	Neurological	27 (7.3%)
	Falls	7 (1.9%)
	Pain	11 (3.0%)
	Miscellaneous	62 (16.7%)

**Table 4:** Accuracy among raters.

	Raters	Accuracy
Rater 1	Emergency department attending	83%
Rater 2	Chat GPT 3.5	84.60%
Rater 3	Chat GPT 4	85.50%

**Table 5:** Cohen’s kappa statistic for agreement between the raters.

Comparison	Kappa	*p*-Value
Rater 1 and Rater 2	0.23	<0.01
Rater 1 and Rater 3	0.31	<0.01
Rater 2 and Rater 3	0.701	>0.01

**Table 6:** Interclass Correlation coefficient between 3 raters.

Raters	3
ICC	0.409
*p*-value	<0.01
95% confidence interval	0.346–0.472

**Table 7:** Accuracy of raters compared with gold standard for the cardiovascular and gastrointestinal systems.

Accuracy	Rater 1 (Emergency Department Attending)	Rater 2 (Chat GPT3.5)	Rater 3 (Chat GPT4)
Gastrointestinal system	84%	85.22%	87.50%
Cardiovascular system	77.90%	81.34%	80.23%

**Table 8:** Cohen’s kappa statistic for agreement between the raters for cardiovascular and gastrointestinal systems.

Comparison	Cohen’s Kappa	
	Cardiovascular System	Gastrointestinal System
Rater 1 and Rater 2	0.32	0.17
Rater 1 and Rater 3	0.51	0.12
Rater 2 and Rater 3	0.74	0.71

**Table 9:** Fleiss’ kappa statistic for agreement between multiple raters for cardiovascular and gastrointestinal systems.

	Fleiss’ Kappa	
	Cardiovascular System	Gastrointestinal System
Rater 1 vs. Rater 2 vs. Rater 3	0.52	0.32

## Data Availability

Data is unavailable due to privacy or ethical restrictions.

## Abbreviations

The following abbreviations are used in this manuscript:

USMLE	United States Medical Licensing Exam
ICC	Intraclass Correlation Coefficient
